# A case of pulmonary tumor embolism syndrome with thrombus in transit

**DOI:** 10.1016/j.rmcr.2023.101896

**Published:** 2023-07-20

**Authors:** Alexandra Fuher, Esther de Boer, Peter Hountras

**Affiliations:** Division of Pulmonary Sciences and Critical Care Medicine, Department of Medicine, University of Colorado Anschutz Medical Campus, Aurora, CO, USA

**Keywords:** Pulmonary tumor embolism syndrome, Percutaneous aspiration thrombectomy, Pulmonary vascular disease

## Abstract

The incidence of pulmonary tumor embolism in patients with solid tumors is estimated to be between 3% and 26% yet is rarely diagnosed. In this case, a 74-year-old male with sarcomatoid variant of urothelial carcinoma and recently diagnosed left renal vein thrombus treated with low-molecular-weight-heparin, presented to the emergency department with acute syncope and dyspnea. He was found to have CT imaging of segmental and subsegmental arterial filling defects, a right atrial filling defect concerning for thrombus in transit and was diagnosed with pulmonary tumor embolism syndrome. The patient was treated with aspiration thrombectomy, with pathology demonstrating sarcomatoid urothelial carcinoma cells. He was initiated on a combination of gemcitabine plus carboplatin to decrease the tumor burden. While pulmonary tumor embolism syndrome is associated with a poor prognosis, prompt diagnosis and initiation of cancer-specific therapies can significantly improve survival.

## 1 Background

The incidence of acute pulmonary thromboembolism ranges from 0.13% to 8.65% in cancer patients [1]. The diagnosis of pulmonary tumor embolism syndrome is often overlooked as the clinical and radiological presentation can be difficult to distinguish from pulmonary thromboembolism often resulting in a delay in diagnosis and treatment. Currently, only ∼6% of pulmonary tumor embolism syndromes are diagnosed antemortem. Whereas autopsy studies estimate that the incidence of pulmonary tumor embolism is between 3% and 26% among patients with solid tumors [2]. Although pulmonary tumor embolism syndrome is associated with a poor prognosis, long-term survival can be significantly improved by early diagnosis and prompt initiation of cancer-specific chemotherapy.

## 2 Case presentation

*History:* A 74-year-old male with a medical history of sarcomatoid variant of urothelial carcinoma (T3bN0M0), status post a cystoprostatectomy with neobladder and subsequent radiation eight months prior to presentation, presented to the ED with syncope and dyspnea. These symptoms started acutely on the day of presentation. Symptoms were accompanied by a new oxygen requirement. He also reported a dry cough, weakness, and lightheadedness for the same period. A week prior to admission, the patient was found to have a new left renal mass, a thrombus involving the left renal vein, and inferior vena cava (IVC), and bilateral pulmonary emboli. He underwent a renal biopsy and IVC thrombectomy. The patient was discharged on low-molecular-weight heparin (LMWH) at a dose of 1 mg/kg twice daily. The patient confirmed compliance with his LMWH, denied recent travel or long periods of immobility.

*Physical Examination:* Vital signs at the time of admission included a temperature of 37.2 °C, a heart rate of 96 beats/min, BP of 94/57 mm Hg, an oxygen saturation of 96% on HHFNC (FiO_2_ 40%, flow 50L/min), and a respiratory rate of 22 breaths/min. The patient was in mild distress. He had jugular venous distension to the level of his earlobe with the head of the bed at 40–45°. He had a normal heart rate and rhythm without significant murmur. He had normal lung auscultation. His abdomen was soft and nontender. No lower extremity edema was appreciated.

**Fig. 1:**
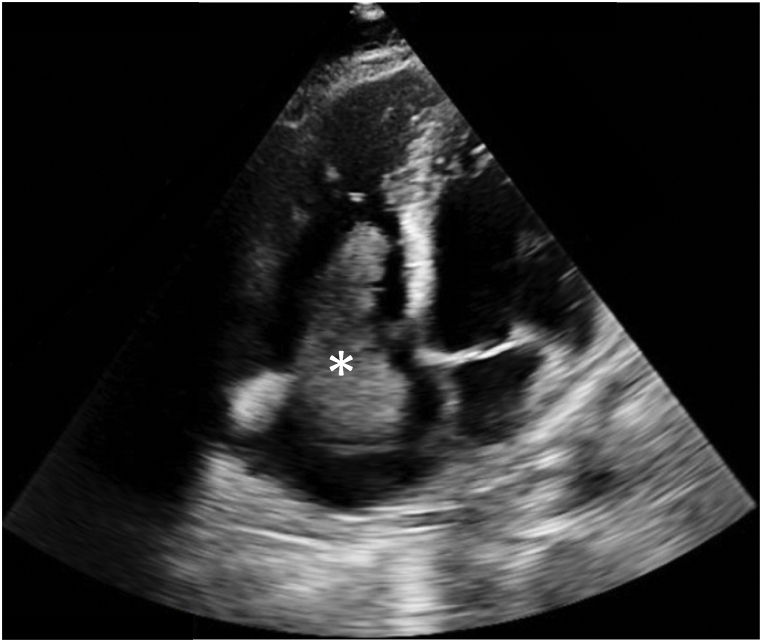
**–** Mobile right atrial thrombus (marked by asterisk) traversing into the right ventricle.

*Diagnostic studies:* Laboratory findings were notable for an elevated pro-brain natriuretic peptide and troponin I-hypersensitivity, 575 pg/mL and 721 ng/L, respectively. The patients complete blood count showed a normocytic anemia with a hemoglobin of 8.9 g/dL, stable from prior. This patient had a normal platelet count on admission (212 × 10^9^/L). Routine coagulation work-up at the time of admission showed a anti-Xa level, INR and prothrombin time of 0.39 U/mL, 1.3 and 15.9 seconds, respectively. On the basic metabolic panel his creatinine was 1.69 mg/dL (increased from ∼1 three months earlier). CT indicated worsened segmental and subsegmental bilateral pulmonary arterial filling defects as well as a filling defect in the right atrium concerning for a thrombus in transit. He had an echocardiogram, which demonstrated right atrial and ventricular dilation with a large (4.2 × 4.5 cm in diameter) mobile right atrial thrombus in transit through the tricuspid valve (Fig. 1). Upon removal and evaluation by pathology, the patient was diagnosed with pulmonary tumor embolism syndrome with thrombus in transit.

## 3 Discussion

For management of pulmonary tumor thrombus in transit, management options include therapeutic anticoagulation, systemic thrombolysis, catheter directed thrombolysis, surgical removal, and aspiration thrombectomy [3]. There are no established guidelines on when and in whom each technique is indicated. Further, the presence of tumor tissue in the embolism decreases the possible therapeutic options for massive pulmonary embolism as anticoagulation and/or thrombolysis alone is unlikely to be successful.

Systemic thrombolysis using tissue plasminogen activator (tPA) has been described to successfully lyse right atrial thrombi and improve hemodynamics [4]. However, there have been cases of unsuccessful thrombolysis, especially in cases of massive thrombi in which the large size of the thrombus may prevent complete lysis. Data suggest that systemic thrombolysis with tPA can be given when massive tumor thrombus embolism is known or suspected to assist with minimization of inflammatory reaction. However, is unlikely to have any therapeutic effect on the tumor cells present in the embolism causing obstruction and hemodynamic collapse. The increased risk of bleeding from systemic thrombolysis, ranging anywhere from 5% to 20%, must be carefully weighed.

Catheter-directed thrombolysis has the potential to offer the benefits of systemic thrombolysis while, in theory, reducing the risk of bleeding by being a more “targeted therapy” [5]. However, catheter-directed thrombolysis is rarely used for the treatment of intracardiac thrombi, fewer than five cases of using this strategy have been reported.

The primary surgical option used for pulmonary tumor embolism syndrome has been open thrombectomy while on cardiopulmonary bypass [6]. Although a definitive treatment, this is one of the most invasive approaches, requires surgical experience, and difficult to perform in unstable patients.

Percutaneous aspiration thrombectomy is an emerging strategy for the management of intracardiac thrombi and has been a successful strategy for treatment of mobile intracardiac thrombi in several case reports [7]. Advantages include avoidance of a sternotomy as well as the bleeding risks associated with systemic thrombolysis. The procedure requires placement of a 26 Fr sheath to allow entrance of the 18 Fr Angiovac catheter into the right atrium, ventricle, and pulmonary arteries. The procedure requires placement of an additional internal jugular or femoral vein catheter to allow for reperfusion. Once the Angiovac catheter is positioned, the extracorporeal circuit is activated. Blood circulates at rates up to 4L/min while collecting aspirated debris in a filter trap. Downsides to this approach include lack of flexibility of the device to reach thrombi in more challenging positions. Furthermore, this approach requires venovenous extracorporeal membrane oxygenation (ECMO) and can therefore only be performed at ECMO-capable centers. Alternatively, percutaneous mechanical thrombectomy has been approved for clot in transit. While developed more recently, this approach is promising as it avoids the need for extracorporeal filtration or cardiopulmonary bypass [8].

Lastly, a more conservative strategy option includes anticoagulation alone with continued observation and reassessment. Ultimately if there is any concern for pulmonary tumor emboli syndrome it is critical to send thrombi for pathology early, as rapid initiation of chemotherapy to decrease the tumor burden is considered the mainstay of treatment. Although its efficiency remains very low, favorable outcomes can be seen in highly chemosensitive tumors.

## 4 Treatment and outcome

**Fig. 2:**
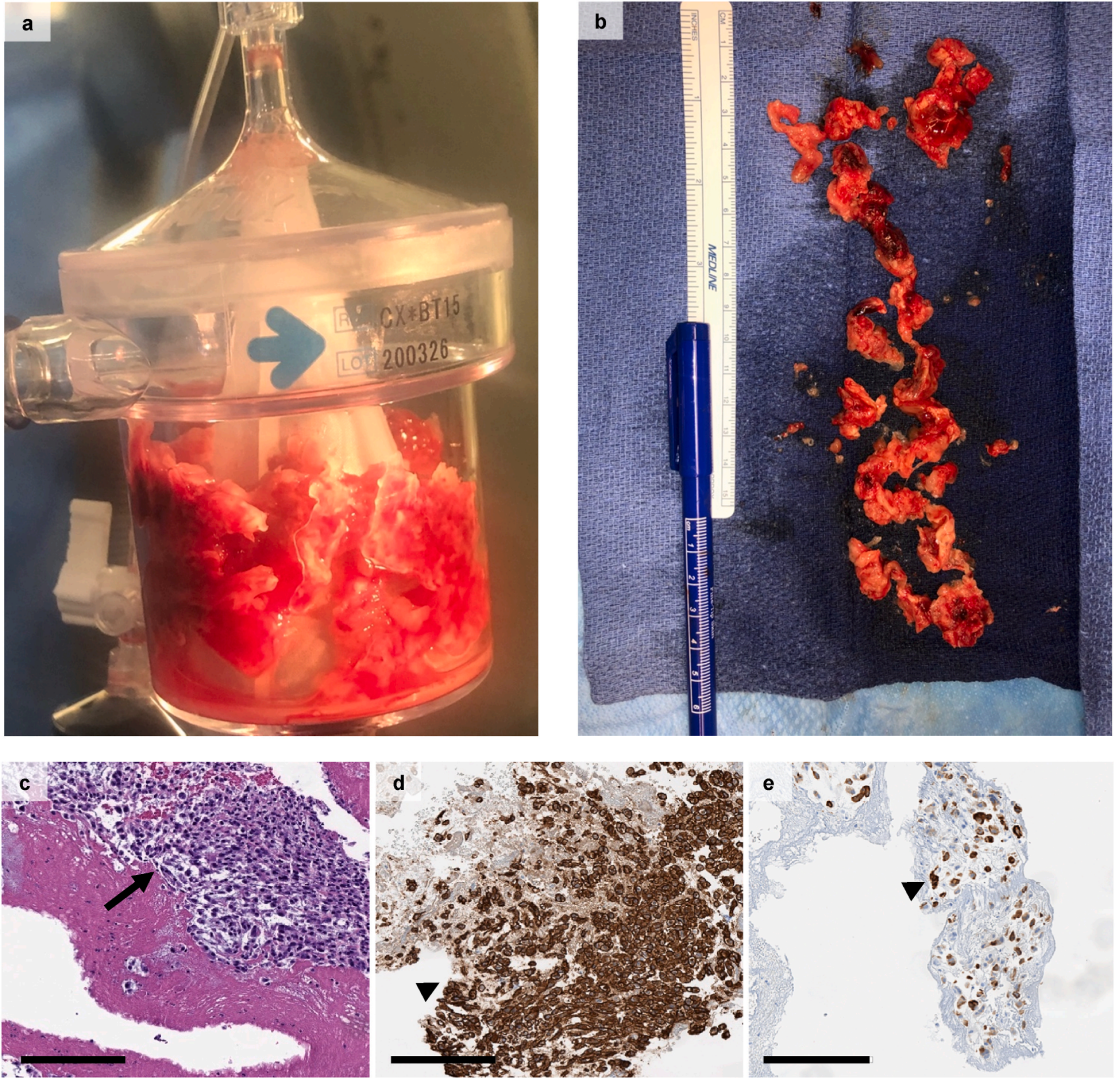
**–** (a-b) Postprocedure removal of right atrial thrombus. (c) Diffuse sheets of sarcomatoid urothelial carcinoma cells (arrow). (d) Tumor cells with diffuse positive immunostaining for carbonic anhydrase IX (arrowhead) a urinary marker for bladder cancer and (e) AE1/AE3 cytokeratin (arrowhead) stains. Scale bar = 200um.

The Pulmonary Embolism Response Team **(**PERT) was consulted for the patient, and the treatment options discussed in this article were considered. It was ultimately decided to pursue aspiration thrombectomy using the Angiovac device. In the case aspiration thrombectomy was unsuccessful the plan was to escalate to surgical right atrial thrombus resection and pulmonary thromboendarterectomy. Aspiration thrombectomy was successful and the tumor thrombus was removed in its entirety (Fig. 2a–b) and sent for pathology and demonstrated sarcomatoid urothelial carcinoma cells (Fig. 2c–e).

Unfortunately, postoperatively the patient developed interval worsening of his right ventricular failure for which he remained intubated and was started on a dobutamine and norepinephrine drip. On postoperative day 1 he was successfully extubated. He required prolonged inotropic support in escalating doses to a max of 12.5 ng/kg/min, likely from continued tumor thrombus showering. On postoperative day 3 he was started on a combination of gemcitabine plus carboplatin to decrease the tumor burden. Due to the worry that this patient had developed chronic thromboembolic pulmonary hypertension limiting his ability to wean from dobutamine, further diagnostic evaluation was done with a ventilation-perfusion (V/Q) scan and right heart catherization (RHC) on postoperative day 14. The V/Q scan demonstrating multiple large, mismatched perfusion defects through multiple segments of the bilateral lungs. No evidence of right heart dysfunction was found during the RHC (central venous pressure, 1 mmHg; pulmonary artery pressure, 30/5 mmHg; pulmonary capillary wedge pressure, 5 mmHg; and cardiac output, 7.38 L/min). On postoperative day 15 the dobutamine was safely discontinued. The patient was initially anticoagulated with an infusion of unfractionated heparin followed by administration of a direct oral anticoagulant. Unfortunately, the patient subsequently developed pyelonephritis and was transitioned home to hospice.

## 5 Learning points

1. The incidence of acute pulmonary thromboembolism ranges from 0.13% to 8.65% in cancer patients.
2. Pulmonary tumor embolism syndrome (with thrombus/clot in transit) is a rare but life-threatening disease entity often overlooked as the clinical and radiological presentation can be difficult to distinguish from pulmonary thromboembolism
3. Several treatment strategies to right atrial tumor emboli in transit are available, including therapeutic anticoagulation, systemic thrombolysis, catheter directed thrombolysis, surgical removal, and aspiration thrombectomy.
4. Aspiration thrombectomy with the Angiovac is a promising therapeutic strategy for thrombectomy.
5. Whatever treatment strategy is chosen, in addition to anticoagulation, it is imperative to treat chemosensitive cancers with chemotherapy to decrease the tumor burden.

## Intellectual property rights assignment or license statement

I, Alexandra Fuher, the Author has the right to grant and does grant on behalf of all authors, an exclusive license and/or a non-exclusive license for contributions from authors who are: i) UK Crown employees; ii) where BMJ has agreed a CC-BY license shall apply, and/or iii) in accordance with the relevant stated license terms for US Federal Government Employees acting in the course of the their employment, on a worldwide basis to the BMJ Publishing Group Ltd (“BMJ”) and its licensees, to permit this Work (as defined in the below license), if accepted, to be published in BMJ Case Reports and any other BMJ products and to exploit all rights, as set out in our license author license.

## Declaration of competing interest

None.
